# Secondary Puerperal Hæmorrhage

**Published:** 1883-03

**Authors:** Paul F. Mundé

**Affiliations:** Professor of Gynæcology and Obstetrics at the New York Policlinic; Professor of Gynæcology at Dartmouth College; Gynæcologist to Mt. Sinai Hospital; Visiting Surgeon to Maternity Hospital, New York


### SECONDARY PUERPERAL HÆMORRHAGE.

By PAUL F. MUNDÉ, M.D., Professor of Gynæcology and Obstetrics at the New York Policlinic ; Professor of Gynæcology at Dartmouth College; Gynæcologist to Mt. Sinai Hospital; Visiting Surgeon to Maternity Hospital, New York.

While the occurrence of more or less alarming uterine hæmorrhage immediately after or during the first twelve hours following delivery is by no means uncommon, and while its causation, prevention, and treatment are thoroughly discussed in all the text-books on obstetrics, and in the current medical press, the subject of metrorrhagia at a later period of the puerperal state, so-called "secondary hæmorrhage," has received comparatively little attention. The majority of obstetric treatises scarcely refer to the possibility that alarming uterine hæmorrhage may occur as late as several weeks after confinement, and only the standard works of Barker, Winckel, Playfair, Spiegelberg and Barnes devote a fair amount of space to this accident. And even these authors, while alluding to individual experience of their own, all refer to the wellnigh exhaustive memoirs of McClintock,* of Dublin, published in 1851, and of Bassett,† of Birmingham, in 1872. Barker and Playfair both remark that this variety of uterine hæmorrhage has not received sufficient notice in the text-books, although, as the latter writer says, it "often gives rise to serious and even fatal results, and is always somwhat obscure in its etiology, and difficult to treat."

As for the medical press, while "post-partum hæmorrhage," that is, the primary and immediate variety, seems a favorite theme, and has even excited the most animated, and at times personal discussions in our obstetrical societies, the, if more rare, not less serious secondary variety, is scarcely ever recorded. It was this very fact, no doubt, which some two years ago induced the learned professor of obstetrics at Indianapolis, Dr. Theophilus Parvin, to write an elaborate essay on this subject, which he read before the American Gynæcological Society at its meeting in Cincinnati in September, 1880, and which is published in Vol. V of the Transactions of that Society. Inasmuch as this admirable series is probably mostly read by specialists, and naturally not as familiar to the general profession as it should be, and the society wishes it were, Dr. Parvin's paper has possibly escaped the notice of many of my readers, as, indeed, I had entirely forgotten its existence (not having been at the meeting where it was read), until after this article was written, when my attention was called to it by a friend. These circumstances, and the comparative rarity and gravity of the accident, are, in my opinion, sufficient reason for the report of the following case, which pre-

---

* Secondary hæmorrhage after parturition.—*Dublin Jour. Med. Sci.*, May, 1851.

† *Brit. Med Jour.*, 1872.

sents, in addition, certain peculiarities of special interest, not referred to by Dr. Parvin.

On August 2, 1882, about noon, I was sent for in great haste by Dr. S. Kohn, to see a lady who was said to be in great danger from uterine hæmorrhage. On arriving at the house, I learned from the doctor the following history: Mrs. C. G., twenty-five years of age, mother of three children, had always been in robust health. Was taken in labor on July 16 with her fourth child. Labor progressed very slowly, in spite of severe pains, until the head almost rested upon the perineum, completely filling the pelvic cavity. As no advance was made, and labor had already lasted twenty-one hours, the forceps were applied, but slipped off twice. An hour later, the forceps having again been attempted with the same result, the cranium was opened with scissors, and the forceps were again applied, and again refused to hold. The cephalotribe was then employed, and the head delivered; the remainder of the child was easily extracted. The cause of the difficulty proved to be hydrocephalus. Hæmorrhage was quite profuse, but soon ceased. The placenta was adherent to the right side of the fundus, and required complete separation by the hand. Care was taken to avoid leaving any fragments behind. Two fluid drachms of ergot were administered by the mouth, and all hæmorrhage ceased. On examination, it was found that the anterior lip of the cervix was quite badly torn.

The patient felt well the next day, and appeared to be doing finely for the next six days, although the temperature and pulse kept at 101.5° to 102° and 120 respectively. The lochia were fetid from the third day on; and the uterine injections washed away numerous small shreds and coagula, until the lochia lost their offensive odor.

On July 28, however, the eleventh day, the lochia again became offensive, but the odor was different from that noticed at first; and the discharge was more scanty, serous, with admixture of a reddish-black fluid, like that discharged from under a slough in any part of the body. The temperature now reached 103° for one hour, and then fell to 102°, remaining at 101° to 102° night and morning for several days. On the thirteenth day, July 30, the temperature was 100° in the morning; 101° in the evening. The patient felt quite well, sat up in bed a little every day, and read the paper. Appetite for solid food absent, but milk, soup, and wine taken in fair quantities. The bladder failed to do its duty, and the catheter was required twice daily. Probably it had been bruised during the long labor and delivery. On the sixteenth day, August 2, at five o'clock in the morning, a profuse hæmorrhage began, which, when the doctor saw the patient at 9:30 o'clock, had almost exsanguinated the patient. Her friends had attached little importance to the flow at that late day, and did not send for him until her weak condition had begun to alarm them. He found her lying in a pool of blood, and immediate interference imperative. The bleeding was arrested for a moment by a hot water intra-uteride injection, but soon recommenced; was again checked by hot injection and tamponade of the vagina, and I was sent for. When I arrived, I found in attendance Drs. S. Kohn, C. A. von Ramdohr, and J. H. Hillyer. The physician who had confined the lady, Dr. Julius Bopp, was not present.

I found the patient with low head, perfectly pallid; face, hands and feet cold and clammy; pulse 120 and very faint; consciousness unimpaired. She merely complained of feeling very weak. On palpating the abdomen, I felt the fundus on a level with the umbilicus, irregular in outline, the right horn extending several inches above the navel. Moderate tenderness. The tampons protruded from the vulva, and were matted together by a moderate amount of bright-red coagulated blood. There was a somewhat offensive odor about the vulva. In spite of the weak condition of the patient, it seemed advisable to remove the tampons and endeavor to arrest the hæmorrhage permanently, which evidently had ceased but temporarily. So long as the uterus was distended by coagula, as at present, no permanent cessation of the bleeding could be expected, and the decomposition of the coagula would but add to the danger by producing septic infection. After preparing fresh carbolized tampons, procuring a few ounces of pure tr. iodine, and a fountain syringe filled with very hot carbolized water, I rapidly removed the tampons, and at once passed my hand into the dilated vagina, and through it into the distended uterine cavity. The latter I found filled with soft coagula, which I speedily removed to the amount of several handfuls. They were exceedingly offensive, dark-colored, and largely mixed with shreds of decidua. After clearing out the uterus, I found its internal surface very soft, pulpy, and the mucous membrane apparently very much thickened. Near the fundus, the finger easily entered the tissue, and the sensation given was exactly as though the opposing surfaces of endometrium had become adherent, and were separated by my finger. I had felt such a condition but once before, in a case of septic endometritis seen in consultation last spring, which terminated fatally. The endometrium was quite smooth, and there was no evidence of retention of placental fragments. Each horn of the uterus was separated from the main cavity by a circular band, the right horn being the larger. After rapidly cleaning out the uterine cavity, wherein caution was necessary in order to avoid injuring, perhaps perforating, the pulpy wall of the organ, I introduced a long metal tube and washed it out with carbolized water from a fountain syringe, the water being as hot as my hand could bear. The patient's sensibilities were so low in her exhausted condition, that she did not complain of the heat. I then introduced a large cylindrical speculum, through it the tip of a long cervical syringe, and injected half an ounce of pure simple tr. iodine into the uterine cavity, using some force in order to ensure the thorough distribution of the iodine. As it flowed into the speculum I mopped it up with cotton, and then packed the vagina with cotton tampons joined by a cord, and withdrew the speculum. Had the patient not been so weak, I should have applied the iodine and the tampons through Sims' speculum, and perhaps have then tamponed the cavity of the uterus itself. But as it was, the tampons were applied merely as a safeguard against further hæmorrhage in case the iodine failed to check it, and I ordered them to be removed in six hours. The patient's extreme weakness made it imperative that she should under no circumstances lose another drop of blood.

The injection of iodine gave no pain whatever, nor was it followed by shock. But the pulse now became so faint that six hypodermics of brandy were given, and ten drops of aromatic spirits of ammonia, five drops of spirits of camphor, and a teaspoonful of brandy, were ordered in ice-water every half-hour. As an additional safeguard against hæmorrhage, a hypodermic syringeful of Squibb's fl. extr. of ergot was injected into the subcutaneous cellular tissue of the abdomen, and an ice-bag placed over the uterus. I was so afraid of a return of the hæmorrhage, that I preferred to risk the possible depressing effects of the ergot on the patient, even in her weak state, rather than to omit any reasonable precaution against hæmorrhage. A bottle of hot water was also placed at the feet.

I then left the patient, with directions, after removal of the tampons, to inject the uterine cavity very gently with tepid carbolized water, as a preventive against the decomposition of the iodine coagula.

The next morning I received a telegram informing me that the patient had rallied. On visiting her twenty-four hours after the treatment already described, I found her with a circular flush on each cheek, bright eyes; pulse 120 and bounding; temp. in axilla 101.5°. There had been no more hæmorrhage, although the uterus had been washed out several times.

The doctor informed me that soon after my departure on the previous day the patient went into so profound a collapse that he thought she would not rally; she also vomited freely. After a few hours, however, she recovered, and he expressed himself as hopeful of her recovery. I told him that I could but repeat my conviction expressed the previous day, that if she rallied from the hæmorrhage I did not fear its return, but that I very much apprehended the issue of the septic endometritis from which the patient seemed to be suffering. In this opinion I was confirmed by the hectic flush and the peculiar sweetish odor about the patient. I directed tepid injections of a dilute solution (1-6 per cent.) of permanganate of potash into the uterus every three hours, more or less, according to the offensiveness of the discharge; further, salicylate of soda in capsules, ten grains every two hours, if borne by the stomach, in case the temperature should rise above 102°. Otherwise. stimulants *ad lib.* My prognosis was unfavorable.

As I was attending a case of confinement, I begged to be excused from further charge of the case, merely signifying my willingness to be of assistance at any future time if wanted. The remainder of the history I owe to the kindness of the attending physician, Dr. Kohn.

Beginning immediately after the permanent arrest of the hæmorrhage, every means was employed to replace the blood lost, and enemata of meat extract, brandy, and warm salt water were given hourly, as well as frequent hypodermics of brandy. The stomach could retain nothing, even iced champagne being rejected. At 3:15 P. M., temp. 101°; at 10:30 P. M., 105°; pulse 132. Reaction had now taken place.

Carbolized intra-uterine injections were employed every three hours.

Aug. 4—Temp., 7:30 A. M., 101.3°; 10:30 A. M., 102.3°; 4:30 P. M., 103.4°.

Aug. 5—Intra-uterine injections with sulphate of quinine.

Morning—Temp., 101.3°. Evening—Temp., 102.5°.

Aug. 6—3:30 A. M., 102°; 7:30 A. M., 100.5°; 9 A. M., 100.1°; 12 M., 101.1°; 8 P. M., 102.1°. Pulse, 114.

Nourishment and brandy taken in good quantity. Sensorium clear. Bowels moved twice after enema. Since yesterday lochia have changed from their putrid to a more natural odor, and appear merely purulent.

A circumscribed swelling is also noticeable to the left of the umbilicus, which Dr. Bopp believes to be an exudation into the broad ligament. An ice-bag was applied. Dr. Kohn thinks this may be an effort of nature to check the disintegrating process going on within the uterus.

Aug. 6—10 P. M. Patient sleeping quietly after an intra-uterine irrigation and an egg-nogg.

Aug. 8—Temp., 100°; pulse 100 to 110. Lochia profuse and purulent; swelling in left side disappearing. General depression and hectic facies have given way to a feeling of comfort and contentment. Uterus syringed several times daily with a solution of sulph. quinine, ʒi to the quart.

The patient made a very slow recovery, it being fully five weeks after the hæmorrhage, as the doctor informed me, before complete convalescence was established. Only the most unremitting care and constant irrigation of the uterus with sol. potass. permang., with stimulants and tonics, finally succeeded in saving the patient's life. The offensive lochia continued for several weeks after the hæmorrhage.

There are several points of interest in this case which I will review in detail.

1. *The cause of the hæmorrhage*—The conditions which may produce secondary uterine hæmorrhage are enumerated by Barker and Playfair as follows: Constitutional: Hemophilia; mental emotion; functional disease of liver (?); incautious use of stimulants; sudden assumption of the erect position. Local: Irregular and inefficient contraction of the uterus; clots in the uterine cavity; portions of retained placenta or membranes; retroflexion of the uterus; laceration of the vagina or vulva; laceration or erosion of the cervix; ("inflammatory ulceration of cervix" Bennett); malignant disease of cervix; pelvic cellulitis; inversion of the uterus; premature sexual intercourse; loaded rectum.

To these I would add one cause which these two authorities do not mention, but which Winckel speaks of, and which I have repatedly seen as the reason for secondary internal hæmorrhage during the first forty-eight hours after labor, namely, the distended urinary bladder. Uterine contraction is interfered with by the enlarged bladder, the uterine cavity fills with blood, and the fundus is found pushed to one side, and even as high as midway between umbilicus and diaphragm. A case is mentioned by Winckel, in which a similar result was produced by a recently developed ovarian cyst, which dragged the uterus up and interfered with its contraction. Profuse hæmorrhage occurred on the third day.

Forcible straining during defecation may also dislodge newly formed thrombi at the placental site. And four cases are mentioned by Winckel in which secondary hæmorrhage occurred during a chill. It is quite plausible that the form of malarial infection known as a "congestive chill" may, by the powerful determination of blood to the internal organs, produce a uterine hæmorrhage even at a late day after confinement. Indeed, Playfair cites Saboia as authority that in Brazil, secondary puerperal hæmorrhage is a common symptom of malarial poisoning, and can only be cured by change of air and the free use of quinine. And Barker, in his well-known paper on "Puerperal Malarial Fever," * reports five cases in which secondary hæmorrhage

* *Amer. Jour. Obst.*, April, 1880.

appeared after the twelfth day, apparently as the result of malarial fever; in two cases the hæmorrhage was alarming. Hanks also reports a case of serious secondary hæmorrhage from the same cause.*

General febrile disturbances (exanthemata, typhoid fever, rheumatism, pneumonia, etc.,) occurring during childbed, may, by means of the greater circulatory activity existing at such times, also cause secondary metrorrhagia.

A cause upon which great stress is laid by Winckel is too early getting up; at least sixteen per cent. of the lying-in women under his care lost blood (in small amount, of course) on the ninth day, soon after leaving their beds for the first time. Still another cause, in which I am particularly interested in this instance, is also spoken of by Winckel, and, so far as I can find, by him alone, and that is, diseases of the inner surface of the uterus, chiefly endometritis. This, Winckel says, is one of the rarer causes of secondary hæmorrhage, which may be due either to the congestion of the endometrium, or (especially in the diphtheritic form) to erosion of the already occluded vessels of the placental site, by sloughing, I presume.

When I was asked by the gentleman first attending the present case, what I thought to be the origin of the hæmorrhage, I replied that the patient seemed to me to be unquestionably suffering from septic endometritis, with a decided hyperplasia of the endometrium, a species of fungoid proliferation; the constant rise of temperature during the week, with the offensive lochia, seemed to speak for this assumption. The bleeding I believed to be due to a superficial sloughing of more or less of the surface of the hyperplastic endometrium. At that time I was not aware that puerperal endometritis is cited as one of the causes of secondary hæmorrhage. I now think it quite possible that a deeper slough, probat the placental site, took place, and that the newly formed thrombi were dislodged. This supposition seems more plausible than that of a universal oozing, since Spiegelberg says that septic endometritis, by its obliteration of the superficial vessels, prevents secondary hæmorrhage. I presume we have all seen the newly

* *Am. Jour. Obst.*, Jan., 1880.

formed decidua in post-mortems from septic endometritis changed into a pulpy, fibrinous mass.

Of course, this view of mine could have been confirmed only by an autopsy; but I can positively assert that no fragment of placenta remained in the uterus, that no deep fissure in the uterine wall existed, and that the endometrium was perfectly smooth. If I am correct, we must consider secondary hæmorrhage a possible additional danger to be apprehended from, and guarded against in, puerperal septic endometritis, a disease surely quite serious enough by itself.

2. *The date of the hæmorrhage after delivery.*—The time at which secondary hæmorrhage is liable to occur varies greatly in accordance with the character of the labor, the care taken of the third stage, the precautions employed during childbed, and accidental circumstances. As a rule, is may be assumed that the more thoroughly and carefully the contraction of the uterus has been assured after delivery of the placenta, and its relaxation guarded against, for some hours at least, the less likely is early secondary hæmorrhage, that is, within the first forty-eight hours, to occur. In institutions where stringent rules exist as to the supervision of the uterus after delivery, and the firm contraction of the organ is insisted upon before the attendant leaves the patient, it is a rare thing to find the uterus distended with blood after the first twelve hours have elapsed. Thus, Winckel cites but three cases out of fifty in which the hæmorrhage occurred during the first three days after delivery. The same proportion will be found in the practices of those physicians who follow the rules referred to, chief among which are the maintenance of firm contraction of the uterus by gentle friction of the abdomen for at least half an hour after the expulsion of the placenta, and the administration of ergot (generally one drachm of the fl. extr. suffices) immediately after the birth of the child. If these rules are neglected, and chiefly if friction is not kept up long enough until permanent contraction is assured, in a few hours the uterus again relaxes, blood oozes from the placento-uterine sinuses, and at the next visit the physician finds himself obliged to express a coagulum of greater or less size, if nature's provision, the after-pains, has not forestalled him. In a recent case which I saw in consultation for an excessive laceration of the perineum, about forty hours after delivery, I found a full bladder and a uterus distended by a dark, soft coagulum of about the size of a fist.

Between the third and tenth days Winckel found eighteen cases of hæmorrhage, for the most part of moderate degree. This frequency must be partly attributed to the exertion of getting up, since most of the women left their beds on the tenth day. Of the whole fifty cases, in twenty-six the hæmorrhage occurred after the tenth day. This last proportion is unusually large, and I am at a loss to account for it, since Winckel omits to give particulars of these cases. As a rule, hæmorrhage occurring or continuing after the woman leaves her bed is due to retention of some small fragment of placenta (chiefly after abortion), or to deficient involution of the uterus, or occasionally to sloughing of the endometrium. I have already stated that, in my opinion, the hæmorrhage in the present case was due to the last-mentioned cause. Bassett * mentions four cases in which the bleeding was due to retention of portions of the placenta or membranes, and came on the tenth, twelfth, fourteenth, and thirty-second days; Barker refers to instances as late as the fifth or sixth week after delivery, and Helfer speaks of one during the fourth week. The oozing which is so frequently found as a consequence of subinvolution and deficient contraction of the uterus, especially after abortion, and after normal labors in flabby, atonic, anæmic women, may last for months, and only ceases after general tonic and astriagent local treatment. In these cases the return of the first menstrual epoch is frequently marked by an unusually profuse flow.

Laceration of the cervix during the recent labor exerts an unquestionable influence in prolonging the oozing from the uterus, both by means of interference with involution and through direct hæmorrhage from the rent itself.

3. *The diagnosis of secondary uterine hæmorrhage* need be mentioned only to point out that in the early cases the outlines of the uterus should always be clearly defined, since the hæmorrhage may be wholly internal, and be suspected only by the pallor or collapse of the patient. At a later date, when the uterine cavity is

* *British Med. Jour.*, 1872.

smaller, the discharge of blood from the vagina of course reveals the nature of the case. The cause of the hæmorrhage, however, may not be so easily ascertained, especially in the later cases, when it may be necessary to dilate the uterine cavity by tents or rubber tubes in order to make the diagnosis by introduction of the finger.

4. *The significance of secondary hæmorrhage* depends partly upon the amount of blood lost and escaping, and partly on the origin of the flow. The sooner the patient is seen and the flow controlled, of course, the less dangerous the case. Hæmorrhage depending on mere temporary atony of the uterus, which can ordinarly be checked at once by friction, compression, and styptics, is evidently less serious than if due to sloughing of the placento-uterine thrombi or bursting of an ectatic vein. While Spiegelberg, who speaks of having seen one serious case of hæmorrhage on the fourth day from simple atony of the uterus, and a number of other severe hæmorrhages from retention of portions of the secundines, did not lose a case, Bassett had two deaths out of thirteen cases, and McClintock collected six fatal cases. Mme. La Chapelle mentions a case of death on the eighth day from internal hæmorrhage and retention of the clot. Barker relates an instance of almost fatal hæmorrhage on the second day from mental emotion, and McClintock one on the twelfth day from taking a dose of brandy while sitting up for the first time, and another on the eighth day from mental excitement. In my case, there can be no doubt that even a very slight additional hæmorrhage would have proved fatal. The occurrence of serious or fatal hæmorrhage at a later date than the fourteenth day after delivery is certainly very rare. The chief evil consequences of protracted secondary hæmorrhage, metrorrhagia lochialis, as Breisky * calls it, is the debilitating effect on the woman, and subsequent uterine disease of some form or other.

5. *The means employed to check the hæmorrhage.*—The injection of pure tincture of iodine into the uterine cavity as a hermostatic after delivery has been recommended by Dupierris and Lente, and practiced by many others in occasional cases; the

* Breisky; Treatment of Puerperal Hæmorrhage, *Volkmann's Klin. Vorträge*, No. 14, 1871, p. 108.

excellent effects of the agent in non-puerperal hæmorrhage have been warmly praised by Thomas Addis Emmet, whose example has been followed by many of us. I am not aware that iodine has been used as an injection in secondary hæmorrhage, but there is no reason why its styptic effects should be less pronounced then than immediately after delivery. Besides, it is an excellent disinfectant, which was of importance in the present case. It does not form hard coagula, as does the tr. of perchloride of iron, which coagula are liable to decompose and give rise to septic infection, or by their presence excite fresh hæmorrhage. I might, it is true, have employed the mixture in equal parts of the tr. ferri perchloridi, or persulphatis and glycerine, according to the practice of Dr. H. P. C. Wilson, whereby the formation of coagula does not take place. And had the hæmorrhage returned, I should undoubtedly have employed this formula. As it happened, the iodine first occurred to me, and proved effectual.

The method of injecting the iodine through a cylindrical speculum is to be recommended, as a means of saving the vagina and vulva from the unavoidable contact with the fluid if the latter is simply injected into the uterus under guidance of the finger. In the present case, finding that I could not pass the nozzle of the syringe quite to the fundus through the speculum, I first inserted the syringe, and then passed the speculum over it.

As regards the application of a tampon after labor, I need scarcely say that it should never be done unless the uterus is so contracted and constantly watched that no internal hæmorrhage can take place. In the present instance, doubtless, the tampon which I removed had caused the accumulation of blood in, and the distension of, the uterus; but it had arrested the external hæmorrhage, which was far more severe than the internal bleeding was likely to be. Besides, at so late a period as sixteen days after delivery, the attending physicians were to a great extent justified in not looking for so easy a distensibility of the uterus. I re-applied the tampon temporarily as a possible safeguard against external hæmorrhage until the patient had had time to rally a little, and with the positive understanding that the fundus uteri should be carefully watched until the tampon was removed.

The hypodermic of ergot was administered more for its specific hermostatic effect, than with any idea of contracting the uterine muscle, which, in its inflamed, pulpy condition, seemed incapable of healthy contraction. In the collapsed condition of the patient, this agent might possibly have been omitted. Still, it did no injury.

It is possible that the hot water injection might have been sufficient to check the bleeding. I thought, however, that it was best to take no chances, but to do the work thoroughly.

As a rule, it may be assumed that the same remedies and measures which are used to check primary uterine hæmorrhage will be effectual in the secondary variety. Even when the hæmorrhage occurs at a late date after delivery, the agents which act chiefly by contracting the uterine muscle, as ergot, viscum album, fl. ext. gossypium, ustilago maidis, etc., will prove useful, as well as the general styptics, like sulphuric acid, cannabis indica, matico, etc. In cases where the hæmorrhage depends on pelvic congestion, saline laxatives, combined with or followed by ergot and sulphate of iron, will be serviceable.

In the earlier periods after delivery, the clearing out of clots from the uterine cavity with the hand, and the excitation of the organ to contraction by external and internal friction, then, if still necessary, the intra-uterine injection of styptics, will be indicated. At a later date, when the uterus has already undergone a certain degree of involution and the cavity is small, the repeated introduction of astringents into it on cotton cones slipped off from applicators, and the firm columnar tamponade of the vagina with flat discs through Sims' speculum, may be required. The latter are more usually cases of protracted bloody lochial discharge, or of constant sanguineous oozing due to subinvolution. The eventual closure of a cervical rent will become imperative after a lapse of at least two months after delivery, if the flow seems to depend on this injury. By the introduction of pure tr. iodine into the uterine cavity on cotton-wrapped applicators, which are immediately withdrawn, about twice a week for several weeks or months, I have in a number of instances achieved a complete cessation of the bloody oozing, a return to regular menstruation if the patient chanced not to be nursing, and a complete involution of the uterus. It has not been my intention to go over the whole ground of secondary puerperal hæmorrage, or to take up separately each of the etiological factors enumerated in the early part of this paper. For a discussion of these, I will refer to the works of Barker, Winckel, and Playfair, or to the original memoir of McClintock, to which, Playfair says, "We owe almost all our knowledge of this condition;" and to the paper by Parvin. I merely desire to add another case to the comparatively few on record, and by pointing out some of its peculiar features, perhaps draw out the experience or opinion of the profession.

Before concluding, I wish to say a word as to the prevention of these hæmorrhages, primary and secondary. Assuming a firm, permanent contraction of the uterus after delivery to be a cardinal principle for the prevention of post-partum hæmorrhage, the following brief rules may be laid down for the management of the third stage of labor, and the early puerperal state:

1. Always keep the supporting and compressing hand on the fundus uteri from the moment the head appears at the vulva until the placenta is expelled.

2. Do not hasten the expulsion of the placenta too much, but by steady, gentle friction of the fundus endeavor to obtain its total spontaneous detachment, the occurrence of which can easily be detected by the uniform firm outline of the contracted uterus on palpitation.

3. Always watch the uterus with the hand, using gentle friction for at least an hour, at intervals, before leaving the patient.

4. Always give ergot (one drachm or more of the fl. extr.) by the mouth immediately after the birth of the child. If chloroform has been given for an operation, or if the labor has been unusually tedious, give the ergot hypodermically, injecting a syringeful of the fl. extract to the depth of one inch, near the umbilicus. It is always advisable to have the syringe filled with ergot, and to see that it is in good working order, before the conclusion of the labor.

5. Always have ice on hand; and if the uterus shows a reluctance to remain contracted, rub the fundus gently with a piece of ice, or insert a cone-shaped piece into the cavity. (As a rule, the injection of hot water, while a powerful styptic, is less agreeable to the patient at this time, and will scarcely be employed unless actual hæmorrhage occurs.)

6. Always make sure by palpation and compression that the uterus contains no coagula, and if such still form, at once express them.

7. Aid in securing permanent contraction of the uterus by an early application of the child to the breasts.

8. If the labor has been tedious or instrumental, or the uterus contracts badly, or the patient is not to nurse the child, or is constitutionally feeble, it is wise to guard against subinvolution and a long continuance of the bloody lochia, by giving the patient a pill containing one or two grains of Squibb's aqueous extract of ergot, two grains of quinine, and one-third of a grain of extract of nux vomica, three times a day for ten days or two weeks, or longer, beginning on the day after delivery. If the stomach is irritable this combination may be given in rectal suppositories, the quantity of ergot being doubled.

9. Before leaving the patient, an equably tight binder should be applied, and if there be tendency to hæmorrhage, a pad should be placed under the binder over the fundus, to secure its steady compression.

10. Examine the cervix and vagina with the finger immediately after delivery, and if there be a laceration of either, be prepared to check possible future oozing by mild astringent injections, or, if need be, applications through the speculum. (Immediate suture, as recommended by some, appears to me rarely feasible, under the conditions as to light, assistants, hæmorrhage, etc., usually accompanying childbirth).

11. Do not allow a lying-in woman to make rapid motions in bed, strain, sit up suddenly or very long, or to leave her bed before the tenth day.

12. Keep watch of the bladder, particularly during the first twenty-four hours, and assure yourself by palpatation and percussion that it is empty and is not interfering with uterine contraction.

13. Instruct the nurse not to insert the nozzle of the syringe too far, or to use too much force in giving the customary cleansing injections, for fear that the cervix may be bruised.

These rules might be extended, but I am confident that if those here given are carefully observed, primary hæmorrhage will be greatly diminished in frequency, and secondary bleeding, particularly of the atonic and subinvolution type, will rarely be met with. Those cases in which accidental causes are at fault, will, of course, be as little under our control as heretofore.—*Archives of Medicine*, Feb. 1883.

